# Correction: Human neuroblastoma cells with acquired resistance to the p53 activator RITA retain functional p53 and sensitivity to other p53 activating agents

**DOI:** 10.1038/s41419-026-08692-3

**Published:** 2026-06-02

**Authors:** M. Michaelis, F. Rothweiler, B. Agha, S. Barth, Y. Voges, N. Löschmann, A. von Deimling, R. Breitling, H. Wilhelm Doerr, F. Rödel, D. Speidel, J. Cinatl

**Affiliations:** 1 Institut für Medizinische Virologie, Klinikum der Goethe-Universität, Paul Ehrlich-Str. 40, 60596 Frankfurt am Main, Germany; 2 blue-drugs GmbH, Komturstr. 3A, 60528 Frankfurt am Main, Germany; 3 Department of Neuropathology, Ruprecht-Karls-University Heidelberg, Im Neuenheimer Feld 220/221, 69120 Heidelberg, Germany; 4 Deutsches Krebsforschungszentrum, Im Neuenheimer Feld 280, 69120 Heidelberg, Germany; 5 Institute of Molecular, Cell and Systems Biology, College of Medical, Veterinary and Life Sciences, Joseph Black Building, B3.10, University of Glasgow, G12 8QQ Glasgow, Scotland United Kingdom; 6 Groningen Bioinformatics Centre, University of Groningen, Nijenborgh 7, 9747 AG Groningen, The Netherlands; 7 Klinik für Strahlentherapie und Onkologie, Klinikum der Goethe-Universität, Theodor-Stern-Kai 7, 60590 Frankfurt am Main, Germany; 8 Children’s Medical Research Institute, 214 Hawkesbury Road, Westmead, 2145 New South Wales Australia; 9 Sydney Medical School, The University of Sydney, Sydney, 2006 New South Wales Australia; 10 Present Address: School of Biosciences, University of Kent, Canterbury, CT2 7NJ UK

Correction to: *Cell Death* & *Disease* 10.1038/cddis.2012.35, published online 05 April 2012

In our article “Human neuroblastoma cells with acquired resistance to the p53 activator RITA retain functional p53 and sensitivity to other p53 activating agents” (full reference provided above), the beta-actin bands in the left panel of Figure 2B was erroneously duplicated and also presented as the beta-actin bands in Figure 1.

Here, we provide an updated Figure 1 with the correct beta-actin bands:
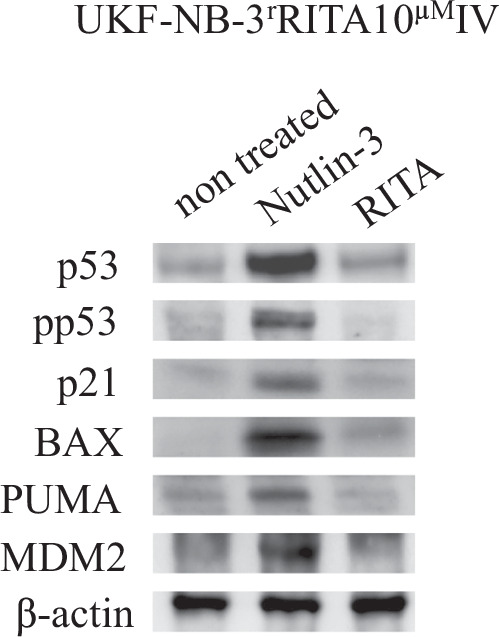


This correction does not affect the study’s conclusions.

All co-authors have reviewed and approved the proposed correction and the revised content.

